# Drug Conjugation Induced Modulation of Structural and Membrane Interaction Features of Cationic Cell-Permeable Peptides

**DOI:** 10.3390/ijms21062197

**Published:** 2020-03-22

**Authors:** Edit Pári, Kata Horváti, Szilvia Bősze, Beáta Biri-Kovács, Bálint Szeder, Ferenc Zsila, Éva Kiss

**Affiliations:** 1 Laboratory of Interfaces and Nanostructures, Institute of Chemistry, Eötvös Loránd University, Budapest 112, P.O. Box 32, 1518 Budapest, Hungary; 2 MTA-ELTE Research Group of Peptide Chemistry, Eötvös Loránd University, Budapest 112, P.O. Box 32, 1518 Budapest, Hungary; khorvati@gmail.com (K.H.); szilvia.bosze@gmail.com (S.B.); beabiri@caesar.elte.hu (B.B.-K.); 3 Institute of Chemistry, Eötvös Loránd University, Budapest 112, P.O. Box 32, 1518 Budapest, Hungary; 4 Research Centre for Natural Sciences, Signal Transduction and Functional Genomics Research Group, Institute of Enzymology, P.O. Box 286, 1519 Budapest, Hungary; szederbalint@gmail.com; 5 Research Centre for Natural Sciences, Research Group of Biomolecular self-assembly, Institute of Materials and Environmental Chemistry P.O. Box 286, 1519 Budapest, Hungary; zsila.ferenc@ttk.mta.hu

**Keywords:** membrane affinity, cell-penetrating peptides, circular dichroism spectroscopy, atomic force microscopy, mycolic acid, Langmuir monolayer, drug–peptide conjugates

## Abstract

Cell-penetrating peptides might have great potential for enhancing the therapeutic effect of drug molecules against such dangerous pathogens as *Mycobacterium tuberculosis* (Mtb), which causes a major health problem worldwide. A set of cationic cell-penetration peptides with various hydrophobicity were selected and synthesized as drug carrier of isoniazid (INH), a first-line antibacterial agent against tuberculosis. Molecular interactions between the peptides and their INH-conjugates with cell-membrane-forming lipid layers composed of DPPC and mycolic acid (a characteristic component of Mtb cell wall) were evaluated, using the Langmuir balance technique. Secondary structure of the INH conjugates was analyzed and compared to that of the native peptides by circular dichroism spectroscopic experiments performed in aqueous and membrane mimetic environment. A correlation was found between the conjugation induced conformational and membrane affinity changes of the INH–peptide conjugates. The degree and mode of interaction were also characterized by AFM imaging of penetrated lipid layers. In vitro biological evaluation was performed with Penetratin and Transportan conjugates. Results showed similar internalization rate into EBC-1 human squamous cell carcinoma, but markedly different subcellular localization and activity on intracellular Mtb.

## 1. Introduction

In the genus *Mycobacterium*, there are three dangerous pathogens for humans: *Mycobacterium tuberculosis* (Mtb), *M. leprae* and *M. lepromatosis* [1]. These pathogens are causative agents of human diseases such as tuberculosis and leprosy. Tuberculosis is still a major public health problem worldwide; it is one of the top 10 causes of death and the leading cause from a single infectious agent (above HIV/AIDS). In 2017, Tuberculosis caused an estimated 1.3 million deaths among HIV-negative people, and there were an additional 300,000 deaths among HIV-positive people [2].

During the therapeutic treatment of infectious diseases, it is important to consider the structural and physicochemical properties of the cell envelope of mycobacteria, in order to allow the access of drug compounds to the cell, for effective targeted drug delivery and treatments. Processes located at the cell envelope of mycobacteria include the protection of the bacterial cell from the environment, transport of solutes and proteins, adhesion to receptors and mechanical resistance [3]. *Mycobacterium* is considered Gram-positive bacterium which possesses a lipid-rich cell envelope [4] composed of a typical plasma membrane and a three-part complex: mycomembrane, arabinogalactan and peptidoglycan. The mycomembrane is a specific barrier composed of mycolic acids, which are the major chemical components (almost 40%) in the cell envelope [5,6,7]. Mycolic acids are exceptionally long chain α-alkyl-β-hydroxyl fatty acids that have different conformational behavior covalently linked to polysaccharide arabinogalactans in the cell wall [3,4,8,9]. The folding and unfolding of mycolic acids can influence its biological function and permeability properties for drugs [7,10]. The Langmuir monolayer is an ideal model to investigate the importance of the lateral organization and the specific functions of lipids, use them for cell mimicking, and allows the systematic study of biomolecule–membrane interactions. [11,12,13]. Hasegawa et al. studied the conformational changes of α-mycolic acid at different surface pressures by using Langmuir–Blodgett technique and atomic force microscopy (AFM) [14]. It was found that α-mycolic acid formed a stable Langmuir monolayer and it is entirely expanded when the surface pressure is high. In experimental model systems, mammalian membranes often mimicked by dipalmitoylphosphatidylcholine (DPPC). This lipid is chosen to represent the most abundant head groups in natural membranes [15]. Chimote et al. used Langmuir monolayers to evaluate the effect of mycolic acid and cord factor (alone and as mixtures) on the surface properties of DPPC layer [7]. It has been found that both mycobacterial lipids inhibit the surface activity of DPPC; furthermore, mycolic acid fluidizes the lung surfactant film, and this can contribute to alveolar collapse in tuberculosis patients.

Various methods have been proposed to inhibit the fission of bacterial cells. Isoniazid (INH) is a first-line antitubercular agent that inhibits the synthesis of mycolic acids. The interaction between antituberculosis drugs (such as INH, rifampicin and ethambutol) and DPPC, which is the main component of the pulmonary surfactant, was investigated by Chimote et al. [16]. Pénzes et al. [17] studied mycolic-acid-containing lipid monolayer models to interpret the membrane affinity of antituberculosis drug conjugates. Change induced in the structure of the phospholipid monolayer by interaction with drug–peptide conjugate was followed by tensiometry and sum-frequency vibrational spectroscopy [18]. Other antibacterial drug candidates and polymers were also applied to determine their membrane affinity and in vitro activity, using lipid mono- and bilayer models [19,20,21]. These studies show the role of the cell envelope in essential processes, and the investigation of its interactions is still an important research field to develop new drugs or drug–conjugates for more effective treatments.

Recently, cationic amphiphilic and antimicrobial peptides (AMPs) have received considerable attention as a possible solution against resistant pathogens. Some of them also showed an anticancer effect by destabilizing the outer membrane of cancer cells and providing selectivity between normal and malignant cells [22]. Cell-penetrating peptides (CPP) have a great pharmaceutical potential as delivery vectors for a wide variety of bioactive cargos. Penetratin [23] and Transportan [24] are among the most-studied CPPs and have the ability to translocate the plasma membrane, delivering drugs to the cytoplasm. The antimicrobial peptide Buforin II (5–21) [25] and Magainin II [26] were isolated from amphibians. Dhvar4 is a synthetic derivative of Histatin, which is a salivary histidine-rich cationic AMP found in the human parotid secretion [27], Crot(1–9,38–42) [28] and CM15 [29], which are hybrid AMPs composed of venom peptides. OT20 [30] is a synthetic oligomer of Tuftsin, a receptor-binding peptide produced by the enzymatic cleavage of the Fc-domain of the heavy chain of immunoglobulin G.

There is a plethora of reports on the investigation of CPPs and AMPs properties [31,32,33], but most of them focus on one or two peptides, without comparison of the members of the given family. In our work, we strived for the comparison of various cationic peptides and their investigation under the same measurement conditions with different techniques. This might be useful to reveal the effect of the composition, polarity, molecular mass and conformational behavior on membrane interactions and transport. For this purpose, a representative set of cationic membrane active peptides was selected based on our previous studies on their cell penetrating ability, hemolytic activity, antimicrobial and cytotoxic effects [34]. Our goal was to identify peptides as promising drug carriers for INH. The selected peptides and their INH conjugated derivatives were synthetized and used to study their interactions with DPPC and DPPC + mycolic acid mixed monolayers employing the Langmuir technique. Circular dichroism spectroscopy (CD) was employed to monitor the secondary structure of peptides and their INH-conjugates in water and also in membrane mimetic-trifluoroethanol-containing media. The influence of conjugation was evaluated and compared for the various AMPs. For further analysis, atomic force microscopy (AFM) was used to image the morphology and structure of peptide-penetrated lipid layer. Cellular uptake was studied on EBC-1 squamous cell carcinoma as lung model cells, and the localization of the peptides was imaged by confocal microscopy. Intracellular killing activity of INH–Penetratin and INH–Transportan was assayed on *Mycobacterium tuberculosis* infected human monocytes.

## 2. Results and Discussion

### 2.1. Peptide Synthesis and Characterization

Cationic membrane active peptides and their INH-derivatives were synthesized in good yields, using previously described methods [35]. Peptides were characterized with their monoisotopic molecular mass (*M*), retention time on a C-18 RP-HPLC column (*R*_t_), estimated hydrophilicity values (*H*) and net charge (*z*) (see Table 1). Peptides are listed according to their retention times, which are the indicator of their polarity. OT20 was found to be the most hydrophilic peptide, while Transportan proved to be the most hydrophobic one in the given set. This order of peptides’ polarity is almost equal to the relative hydrophilicity estimated from the amino acid composition. The conjugation with INH does not change the relative polarity of the peptides, as the *R_t_* values show. It is notable, however, that some increase in the hydrophobicity can be presumed from the slightly increased retention-time values for most of the peptides (except Buforin and Transportan). That might be the result of decreased charge due to INH coupling at the *N*-terminal, although the addition of INH molecule is expected to increase the polarity of the conjugate. 

### 2.2. Interaction of Peptides and INH–Peptide Conjugates with Lipid Monolayers

**Figure 1:**
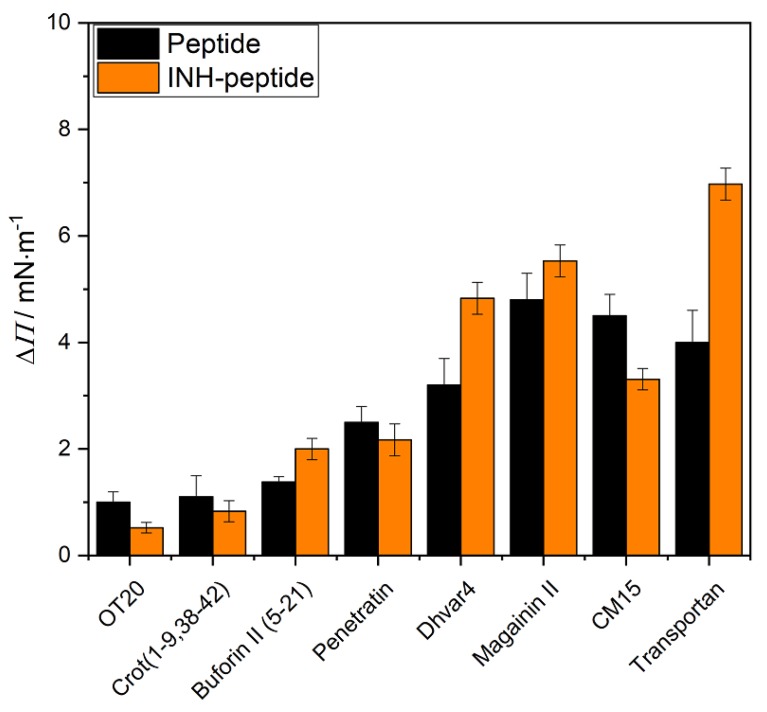
The degree of penetration (Δ*Π*) of the original peptides (black) and their INH conjugated derivatives (orange) into DPPC lipid monolayer after 1 h interaction. (The peptides are presented according to their increasing *R*_t_ values.)

**Figure 2:**
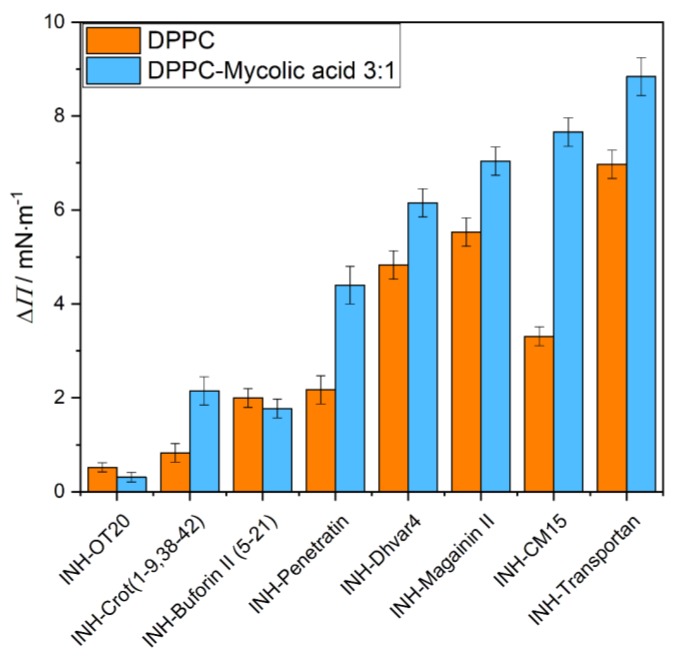
The degree of penetration (Δ*Π*) of isoniazid (INH)-conjugated peptides into DPPC (orange) and DPPC+mycolic acid (3:1 mass ratio) (blue) mixed lipid monolayers after 1 h interaction. (The peptide conjugates are presented according to their increasing *R*_t_ values.)

The peptide–lipid interactions were investigated by the Langmuir technique, and the results of the penetration measurements are presented in Figure 1 and Figure 2. The lipid layer was formed from DPPC and the mixture of DPPC+mycolic acid at ratio of 3:1. The aqueous solution of the peptide or the INH-conjugated peptide was injected below the compressed (20 mN/m) and conditioned (20 min) lipid monolayer after 10 min relaxation time. The final concentration was 2 µM in the aqueous phase. The changes of the surface pressure as a function time was recorded for 1 h. The increase of the surface pressure (Δ*Π*) gives information of the degree of penetration, which is considered as the membrane affinity of the given peptide to the lipid layer.

The penetration results showed that the membrane affinity to DPPC layer is modest (Δ*Π* < 2) in case of OT20, Crot(1–9,38–42) and Buforin II (5–21), but higher for the Penetratin (Figure 1). The other peptides’ membrane affinity then gradually increased. This shows agreement with our previous results, where the membrane affinity order of the peptides almost followed their hydrophobicity [37]. Transportan, CM15 and Magainin are shown to be the most hydrophobic ones in the present set, according to their *R*_t_ (>12) and *H* (< 0) values. Those exhibit significant membrane affinity, exceeding a 4 mN/m increase in surface pressure. The exact order, however, exposes some differences which might indicate that other factors than overall hydrophobicity of the peptide also influence the degree of interaction with the lipid layer.

INH conjugation did not change significantly or decrease membrane affinity of peptides with DPPC monolayer, except in the case of Buforin II (5–21), Dhvar4 and Transportan, where the penetration increased. The coupling of INH to the peptides due to the reduction of charge at the N terminal is expected to increase the hydrophobicity and surface activity character of the conjugate. Therefore, the penetration of the most hydrophobic peptide, Transportan, is further increased for INH–Transportan. The explanation might be similar for the case of Dhvar4, where the INH conjugation induced a relative decrease in polarity counts more because of the lower molecular weight. On the contrary, coupling INH to highly charged hydrophilic peptides probably does not induce important changes in the polar character of the derivatives. That is in correspondence with the slightly decreasing membrane affinity of OT20, Crot(1–9,38–42), Penetratin and CM15 conjugates.

Besides the overall polarity, the conjugation with INH is supposed to induce some structural changes that lead to preferred or unfavored interaction with lipid layers.

The membrane affinity of the INH-conjugated peptides was also characterized by using another membrane model, which is formed from DPPC+mycolic acid (3:1) mixture (Figure 2). By mixing mycolic acid, the monolayer provides a negatively charged layer, which gives the possibility of the electrostatic interactions with the cationic CPPs. This negatively charged environment in the lipid membrane favors the insertion of CPPs [31,38]. Moreover, the incorporation of mycolic acid molecules with long alkyl chains into the lipid layer changes the molecular order and reduces the regular close packing of the DPPC molecules, allowing the interaction with and penetration of the peptides. According to the experimental findings, the membrane affinity significantly increased in all cases except for INH–OT20 and INH–Buforin II (5–21). INH–Transportan and INH–CM15 have the highest interactions with the DPPC+mycolic acid mixed lipid monolayer. 

### 2.3. Circular Dichroism Spectroscopic Evaluation of the Secondary Structure of CPPs/AMPs and Their INH Conjugates

Circular dichroism (CD) spectra recorded in the far-UV region (190–260 nm) provides valuable information on the secondary structure of peptides in a particular medium [39]. In many instances, cationic AMPs/CPPs are substantially unstructured in aqueous solution but acquire helical conformation upon contact with negatively charged cell membranes and also in certain organic solvents, like trifluoroethanol (TFE) [40,41,42]. TFE and related haloalcohols provide a low dielectric, lipomimetic environment which favors and propagates the structuration process of cationic peptides being disordered in water. Membrane-binding-induced folding of cationic peptides into amphipathic helices is a crucial step in their biological actions. Therefore, their structural modifications enhancing helix propensity often result in increased antibacterial and/or hemolytic activity [43,44,45].

**Figure 3:**
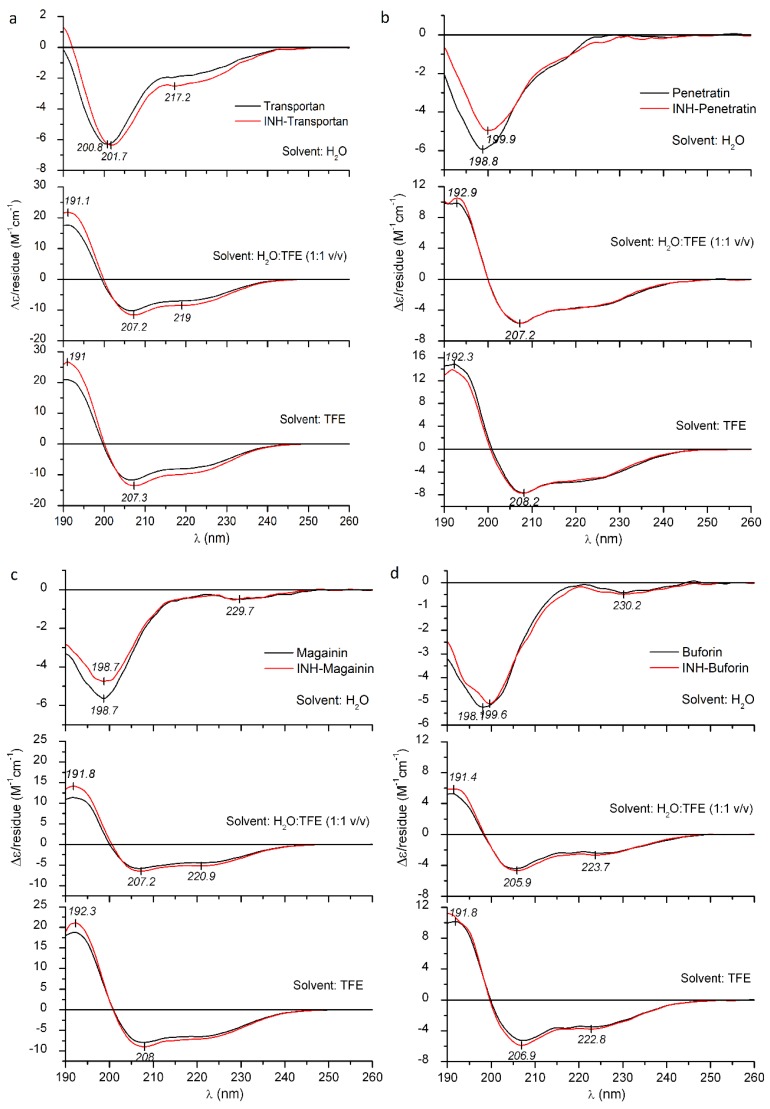
Far-UV CD spectra of Transportan (**a**), Penetratin (**b**), Magainin II (**c**), Buforin II (5–21) (**d**) and their INH conjugates measured in deionized water, TFE and in water:TFE mixture.

**Figure 4:**
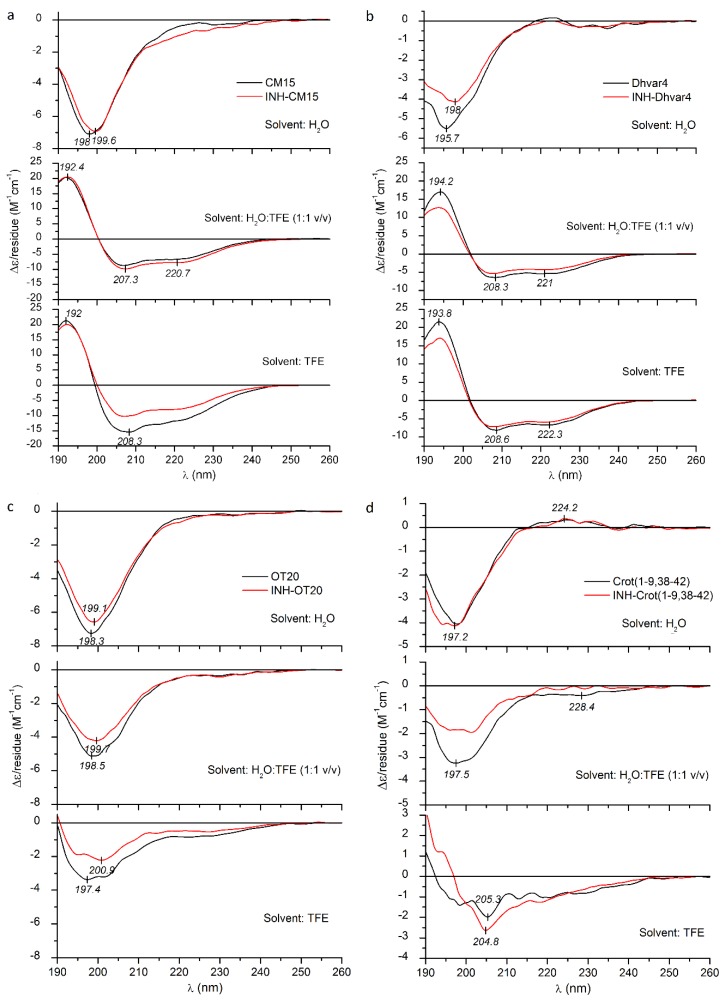
Far-UV CD spectra of CM15 (**a**), Dhvar4 (**b**), OT20 (**c**), Crot(1–9,38–42) (**d**) and their INH conjugates measured in deionized water, TFE and in water:TFE mixture.

All native peptides studied herein exhibit a main negative CD band in water, with a deep minimum, around 196–200 nm (Figure 3 and Figure 4). Such a CD pattern is consistent with the dynamic equilibrium of the mixture of random conformational states. In the presence of TFE, however, a significant amount of helical structure was induced, as evident from the high-intensity positive–negative CD couplet evolved below 210 nm and the pronounced minimum at 220–222 nm, which represent the contribution of the optically active π-π^*^ and n-π^*^ transitions of the peptide bonds arranged along a right-handed helix [39]. Consideration of peak intensities of the CD couplets observed in TFE and water:TFE mixtures permits comparisons of relative α-helical propensities of the peptides. The largest Δ*ε* extrema were observed for CM15, Dhvar4 and Transportan, whereas Penetratin and Magainin II showed somewhat lower values (Figure 3 and Figure 4). Buforin II (5–21) displayed the weakest helical induction with a Δ*ε*_max_ of +10 as measured in TFE (Figure 3d).

It is to be noted that Crot(1–9,38–42) and OT20 behaved differently, since neither of them showed helical conversion upon addition of TFE (Figure 4c,d). For OT20, this result can be explained by the combined occurrence of proline and glycine residues in the sequence that are well-known, potent helix breaker residues [46,47]. Crot(1–9,38–42) lacks proline, but its 14 AA long sequence contains three glycine side chains, creating the highest relative helix breaker frequency among the peptides under study (apart from OT20). Therefore, glycines might be responsible for preventing the canonical α-helical folding of Crot(1–9,38–42).

According to our previous data [48,49,50], small-molecule-binding-induced disorder-to-helix transitions of cationic AMPs are associated with characteristic CD spectral alterations: In the very first phase of the titration with the folding inducer, the negative CD band of the peptide below 210 nm loses intensity, and its λ_min_ is shifted to longer wavelengths. Similar changes were reported for TFE titration of intrinsically disordered peptides [51] and protein regions [52,53]. Taking these results into consideration, the bathochromic shift, as well as the intensity reduction of the main CD peak observed in water, suggests that INH conjugation affects the aqueous conformational equilibrium of all peptides (Figure 3 and Figure 4). Furthermore, as demonstrated by band-intensity increases for some peptide conjugates, this effect is more pronounced in TFE and H_2_O:TFE solution and refers to the moderate enhancement of the α-helical fraction compared to the native form. Such kinds of spectral alterations were obtained for INH–Transportan, INH–Magainin II and INH–Buforin II (Figure 3). The most advanced CD spectral modifications were seen for INH conjugate of Transportan (Figure 3a). At the short-wavelength side of the aqueous spectrum, the initial part of the helix related positive CD peak appeared, and the n-π^*^ CD intensities increased noticeably above 215 nm. Taking into account the positive correlation between peptide helicity and bilayer-disturbing potency [43,44,45], the chiroptical data predict that the INH conjugates bind to lipid membranes with greater affinity compared to the native peptides. Based on the measure of the conjugation-induced spectroscopic changes (Figure 3), an estimation of the relative membrane affinity increment can be proposed (in decreasing order): INH–Transportan, INH–Magainin II, and INH–Buforin II (5–21). Importantly, this prediction is in good correlation with the membrane affinity enhancement of these conjugates displayed in Figure 1. However, the reduced Δ*ε* values of INH–Dhvar4 obtained in TFE and H_2_O:TFE mixture indicate a lower helical propensity (Figure 4b), but its DPPC affinity is larger than that of the parent peptide (Figure 1). In contrast, the reduced CD values above 200 nm in the TFE spectrum of INH–CM15 (Figure 4a) also suggest a lower helicity that is in line with the decreased membrane binding shown in Figure 1. Beyond the impact of the helical conformation, these examples warn to the role of additional INH-conjugation-related physicochemical modifications which may also affect the peptide-membrane interactions.

The presence of helix breaker residues makes it difficult to assess the conjugation-induced structural impact of OT20 and Crot(1–9,38–42) (Figure 4).

### 2.4. Atomic Force Microscopy (AFM)

**Figure 5:**
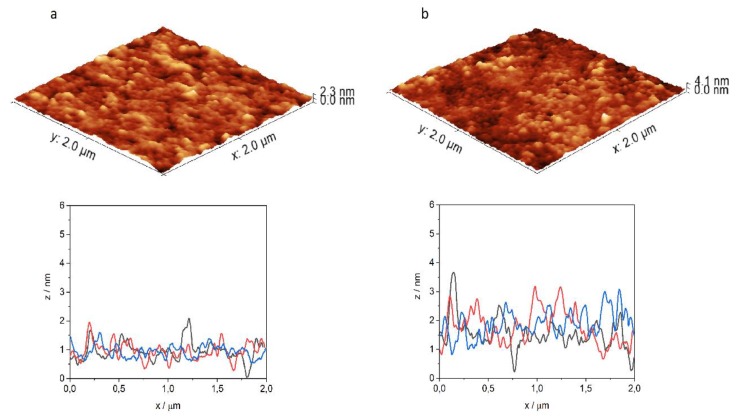
Three-dimensional atomic-force microscopy image of DPPC (**a**) and DPPC+mycolic acid (**b**) lipid layer after 1 h interaction with INH–Penetratin, and characteristic cross-section profiles of the penetrated films.

**Figure 6:**
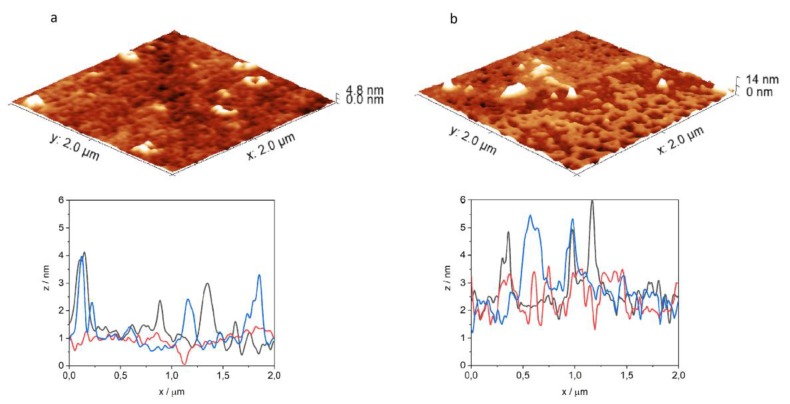
Three-dimensional atomic-force microscopy image of DPPC (**a**) and DPPC+mycolic acid (**b**) lipid layer after 1 h interaction with INH–Transportan, and characteristic cross-section profiles of the penetrated films.

At the end of the membrane affinity measurements, the penetrated monolayers were transferred to a previously cleaned cover glass, to study the structural changes of the membranes by atomic force microscopy (Figure 5 and Figure 6). The surface of the original DPPC and mixed DPPC+mycolic acid monolayer is rather smooth, with a morphology described by typical roughness values presented in Table 2. (*R*_a_ is the average deviation of all points from a mean level over the evaluation area. *R*_q_ (RMS) is the root mean square roughness, while *R*_z_ is the average absolute value of the five highest peaks and the five lowest valleys over the evaluation area, being sensitive to positive or negative extremes of the image.) Well-defined homogenous lipid islands are formed by the ordered lipid molecules in equilibrium with the less-oriented film with smaller surface density as expected from the surface pressure–area isotherm. Comparing to this, the morphology of the penetrated monolayers was changed. The 3D images provide a visual impression on the surface, while the cross-section profiles allow the measurement of the height differences. As examples, for INH–Penetratin and INH–Transportan interaction with DPPC lipid monolayer was found that the homogenous lipid layer was disturbed by the insertion of the peptide-conjugate and several small aggregates, extended protrusions appeared with about 1–3 nm height (Figure 5a and Figure 6a). This morphological change can also be expressed by the roughness factors determined by analyzing the AFM images (Table 2).

It is noticeable that, considering all three parameters, the roughness of the surface increased as a result of its interaction with the peptide conjugates. Similar membrane morphology change was detected previously in the course of interaction of lipid monolayers with cationic, amphiphilic polymers presenting antibacterial properties [21]. 

The result of penetration of INH–Penetratin and INH–Transportan into DPPC+mycolic acid film was presented in Figure 5b and Figure 6b. The disturbance of the order of lipid molecules and the deviation from the smooth surface are even higher, in comparison to the corresponding interaction with DPPC layer. The INH–Transportan forms aggregates as a sort of spherical dots and rings between and within the lipid islands with 3–6 nm height, while in the case of INH–Penetratin, a generally disturbed surface appeared with vertical deviation exceeding 3 nm. The intense disturbance of the mixed lipid layer by interaction with INH–Transportan resulted in the fragmentation of the monolayer. The appearance of aggregated structural units is particularly characteristic for the interaction with the negatively charged DPPC+mycolic acid layer. It has already been shown that such aggregation is the consequence of the proximity of the membrane, and this phenomenon can be triggered by a high concentration of the CPPs on negative phospholipids and induced conformational changes [32]. The electrostatic interactions between cationic peptides and negatively charged lipid layer facilitate the conformational change, and agglomeration of CPPs especially for amphipathic peptides such as Penetratin [31]. 

The significantly enhanced interaction of INH–peptide conjugates with the DPPC+mycolic acid lipid layer, shown by the highly disturbed structure, is in harmony with their high degree of penetration, measured by the increase of surface pressure of the film (Figure 2). It is also in agreement with the CD measurements, since this type of morphology might be the result of enhanced membrane interaction, which involves a membrane-induced increase of the α-helical fraction observed by CD spectroscopy.

### 2.5. Cellular Uptake, Localization and Efficacy against Intracellular Mycobacterium Tuberculosis

**Figure 7:**
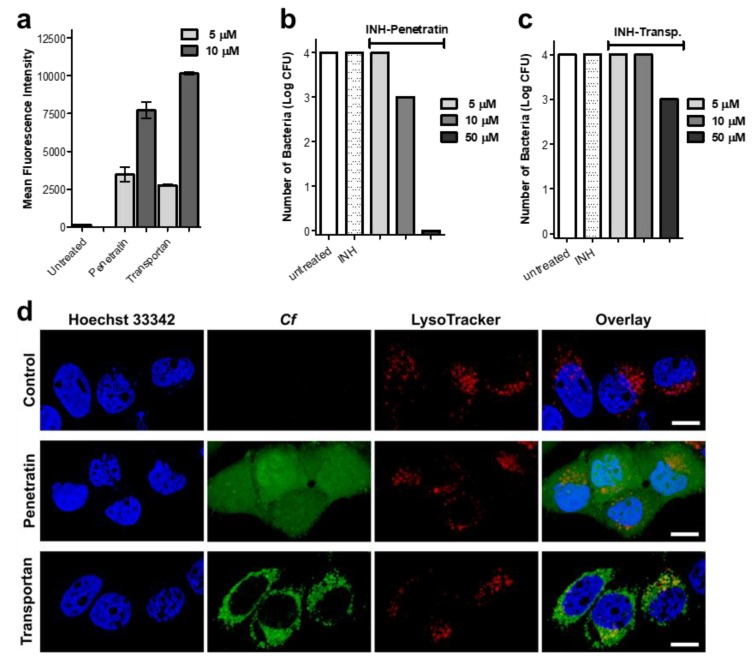
Internalization and intracellular antibacterial activity of the peptides. Cellular uptake of EBC-1 human lung carcinoma cells was measured by flow cytometry after 2 h treatment with 5 and 10 µM peptide concentrations (**a**). Localization of 5(6)-carboxyfluorescein-labeled Penetratin and Transportan in EBC-1 cells was visualized by confocal laser scanning microscopy (**b**). Cells were incubated with *Cf*-labeled peptides (green), lysosomes were stained with LysoTracker Deep Red (red) and nuclei were stained with Hoechst 33342 (blue). The scale bar represents 20 µm. Penetratin and Transportan were conjugated with Isoniazid, and INH–Penetratin and INH–Transportan were assayed against *Mycobacterium tuberculosis*-infected MonoMac-6 human monocytes. After treatment, cells were lysed with SDS, and number of bacteria was enumerated on LJ tubes (**c**,**d**).

For the in vitro biological evaluation, two peptides were selected, namely Penetratin and Transportan, which are the two most-studied cell-penetrating peptides. Internalization of the peptides was measured on EBC-1 human squamous cell carcinoma, which was chosen as a lung cell model. Squamous-cell aggregates are common within the lung tissue associated with *Mycobacterium tuberculosis* (Mtb) infection. Mtb infected host cells (mainly macrophages and other lung tissue cells) play a pivotal role in chronic-tuberculosis-induced carcinogenesis caused by DNA damage, growth factors, proliferative activities, etc. To eliminate the intracellular bacteria from the host cells is crucial for successful therapy. The infection caused mainly by proliferative changes in the lung tissue (mainly in bronchial and alveolar mucosa) that Mtb leaves behind cannot be ignored [54,55]. Therefore, squamous cell culture can be employed as a suitable model to investigate the main features of the carrier peptides. Flow cytometric measurements revealed a concentration-dependent internalization of the peptides. Penetratin and Transportan showed similar internalization potency based on the detected fluorescent intensity measured by flow cytometry (Figure 7a). However, on the confocal microscopy images, markedly different subcellular localization was observed. Penetratin showed diffused intracellular distribution, while in the case of Transportan, mostly vesicular localization was captured (Figure 7b). Besides cytoplasmic localization, Penetratin showed co-localization with lysosomal and nucleus staining, possibly indicating that this compound internalizes not only through the endo-lysosomal pathway, but also through direct cell penetration (Figure 7b). This observation is in line with previous reports on the nuclear translocation of Penetratin [56]. The vast majority of CPPs can be trapped in endosomes, following delivery into the cells. Subsequently, any conjugated cargo might be trapped, as well [57]. Significantly lower intracellular antitubercular activity of Transportan (Figure 7c) might be the consequence of an endosomal escape problem, while the diffuse intracellular delivery makes INH–Penetratin an efficient candidate against intracellular *Mycobacterium tuberculosis* (Figure 7d). 

## 3. Materials and Methods

### 3.1. Peptide Synthesis and Characterization

For peptide synthesis, amino acid derivatives were obtained from Iris Biotech (Germany). The reagents *N,N’*-diisopropylcarbodiimide (DIC), triisopropylsilane (TIS), 1-hydroxybenzotriazole (HOBt), 1,8-diazabicyclo[5.4.0]undec-7-ene (DBU), isoniazid (INH), glyoxylic acid and 5(6)-carboxyfluorescein (*Cf*) were purchased from Sigma-Aldrich (Budapest, Hungary). Fmoc-Rink Amide MBHA resin was from Merck (Budapest, Hungary). Trifluoroacetic acid (TFA), *N,N*-dimethylformamide (DMF), dichloromethane (DCM), diethyl ether and acetonitrile (AcN) were from VWR (Budapest, Hungary).

For the in vitro assays, RPMI-1640 medium, fetal calf serum (FCS) and trypan blue were obtained from Sigma-Aldrich. Trypsin was from Gibco. HPMI buffer (9 mM glucose, 10 mM NaHCO_3_, 119 mM NaCl, 9 mM HEPES, 5 mM KCl, 0.85 mM MgCl_2_, 0.053 mM CaCl_2_, 5 mM Na_2_HPO_4_×2H_2_O, pH = 7.4) was prepared in-house, using components obtained from Sigma-Aldrich (Budapest, Hungary).

#### Synthetic Procedures

Peptides were produced on solid phase (Fmoc-Rink Amide MBHA resin, capacity = 0.67 mmol/g), in an automated peptide synthesizer (Syro-I, Biotage, Uppsala, Sweden), using standard Fmoc/tBu strategy with DIC/HOBt coupling reagents. Isoniazid was conjugated to the *N*-terminus of the peptides by using glyoxylic acid as a linker (the synthesis is detailed in [35]). Fluorescently labeled derivatives were synthesized with the use of 5(6)-carboxyfluorescein (*Cf*) with DIC/HOBt coupling method. Peptides were cleaved from the resin with TFA/H_2_O/TIS (9.5:2.5:2.5 *v*/*v*) mixture (2 h, RT). After filtration, compounds were precipitated in cold diethyl ether, centrifuged (4000 rpm, 5 min) and freeze-dried from water.

RP-HPLC purification was performed on a Thermo Fisher UltiMate 3000 Semiprep HPLC with a Phenomenex Jupiter Proteo C-12 column (250 × 10 mm), using gradient elution, consisting of 0.1% TFA in water (eluent A) and 0.1% TFA in acetonitrile/water = 80/20 (*v*/*v*) (eluent B).

Purified peptides were analyzed by RP-HPLC, on an analytical C-18 column, using gradient elution with the abovementioned eluent A and B (flow rate was 1 mL/min, UV detection at λ = 220 nm). Molecular mass of the peptides was determined on a Thermo Scientific Q Exactive™ Focus Hybrid Quadrupole-Orbitrap™ Mass Spectrometer (ThermoFisher Scientific, Waltham, MA, USA).

### 3.2. Membrane Affinity Measurements

**Figure 8:**
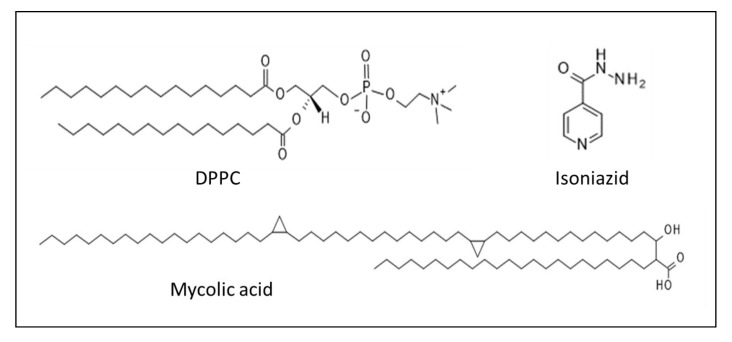
Chemical composition of the drug and lipids applied in Langmuir monolayer experiments

Monomolecular lipid membrane models were used to characterize the interactions between the monolayers and a series of isoniazid (INH)-conjugated peptides. The membrane affinity was investigated by using the Langmuir technique. Surface pressure was recorded with the tensiometric method, with ±0.5 mN/m accuracy, employing previously purified filter papers, such as Wilhelmy plates. The trough was made of Teflon and was equipped with one surface pressure sensor and two polyoxymethylene (POM) barriers. Dichloromethane (VWR Chemicals, analytical purity) and methanol (VWR Chemicals, Reag. Ph. Eur. for HPLC) were used for the cleaning of the troughs and barriers before each measurement. Then, 1,2-Dipalmitoyl-sn-glycero-3-phosphocholine (DPPC) (Avanti Polar Lipids Inc., Alabaster, AL, USA) and its mixture with mycolic acid from *Mycobacterium tuberculosis* (human strain) (98% Sigma-Aldrich) were applied in chloroform (VWR Chemicals, analytical purity), to form the monomolecular lipid layer on the top of the double-distilled water subphase in the Langmuir through. (Molecular structure of isoniazid, DPPC and mycolic acid are presented in Figure 8). The composition of the lipid mixture was a mass ratio of 75w% DPPC and 25w% mycolic acid. Double-distilled water was checked by its conductivity (<5 mS) and surface tension (>72.0 mN/m at 23 ± 0.5 °C) values. After the cleaning procedure, the through was filled with double-distilled water. DPPC or the mixture of DPPC and mycolic acid was dissolved in chloroform to a concentration 0.2 mg/mL. The lipid solution was spread on the subphase. The system was left for 10 min, to evaporate the chloroform, before the compression. Surface pressure–area isotherms were recorded two times before each penetration measurement. The lipid layer was conditioned by barrier adjustment for 20 min, and the aqueous solution of the peptide was injected under the lipid monolayer, after 10 min relaxation time. The peptides were dissolved in double-distilled water, at 200 µM concentration. This peptide solution was injected into the subphase, to achieve 2 µM final concentration. The changes of the surface pressure as a function of time were recorded for 1 h.

### 3.3. Circular Dichroism (CD) Spectroscopic Measurements

Peptide samples were dissolved in deionized water and in TFE at 4–6 µM concentrations. Far-UV CD curves were taken on a JASCO J-715 spectropolarimeter, at 25 ± 0.2 °C, in 0.5 cm path-length rectangular quartz cuvette (Hellma, Plainview, NY, USA). Temperature control was provided by a Peltier thermostat. The CD data were monitored in continuous scanning mode, between 190 and 260 nm, at a rate of 100 nm/min, with a step size of 0.1 nm, response time of 2 s, eight accumulations and 2 nm bandwidth. The CD curves were corrected by spectral contribution of the blank solvent. CD spectra were plotted in mean residue molar CD units (Δε/residue) calculated by the following equation:

Δε = Θ/(32.98*cl*) (1)

where Θ is the measured ellipticity (deg) as a function of wavelength (nm), *c* is the mean residue molar concentration and *l* is the optical path length (cm).

### 3.4. Atomic Force Microscopy Measurements

Penetrated Langmuir monolayers were transferred to a previously cleaned hydrophilic cover glass for atomic microscopy measurements. The cover glasses were cleaned by ultra-cleaning, with the mixture of H_2_O_2_ and H_2_SO_4_, for two hours. After washing and drying, they were suitable for the deposition. The structure of the monolayers was investigated by Nanosurf Flex-Axiom (Liestal, Switzerland) atomic force microscope system, operating in contact mode. Silicon cantilever ContGD-G (BudgetSensors) was used, with a tip curvature radius less than 10 nm, an average force constant of 0.2 N/m, resonance frequency of 13 kHz and with gold reflective coating. Each sample was imaged in 2 × 2 µm^2^ areas, at 10 randomly selected locations. The image analysis was performed by Gwyddion 2.50 program.

### 3.5. In Vitro Assays

#### 3.5.1. Cellular Uptake and Localization of the Peptides

Internalization of *Cf*-labeled Penetratin and Transportan was measured on EBC-1 (squamous cell carcinoma, origin: bronchi) [58,59]. Cells were maintained as an adherent culture in DMEM medium supplemented with 10% heat-inactivated fetal calf serum (FCS) l-glutamine (2 mM) and gentamicin (35 μM), at 37 °C, in a humidified atmosphere containing 5% CO_2_. For the assay, cells were treated with peptides at 5 and 10 µM final concentration and were incubated for 2 h. After centrifugation (1000 rpm, 5 min) and washing with serum-free DMEM medium, supernatant was removed, and 100 μL 0.25% trypsin was added to the cells. After 10 min incubation, 0.8 mL 10% FCS/HPMI medium was added, and then cells were washed and resuspended in 0.25 mL HPMI medium. The intracellular fluorescence intensity of the cells was measured on a BD LSR II flow cytometer (BD Biosciences, San Jose, CA, USA) on channel FITC (emission at λ = 505 nm), and data were analyzed with FACSDiva 5.0 software. All measurements were performed in triplicates, and the mean fluorescent intensity, together with standard error of the mean (SEM), was graphically presented.

Localization of the peptides was visualized by confocal microscopy. EBC-1 cells were seeded (10^5^ cells/well) one day prior to treatment, on cover glass containing 24-well plates (Sarstedt, Nümbrecht, Germany). Lysosomes were stained by LysoTracker™ Deep Red (Invitrogen, Carlsbad, CA, USA) for 30 min, followed by incubation with *Cf*-labeled Penetratin and Transportan for 60 min. Subsequently, nuclei were stained by Hoechst 33342 solution (Thermo Scientific, Waltham, MA, USA). After each step, cells were washed three times with serum-free medium. Cells were fixed by 4% paraformaldehyde for 15 min and mounted to microscopy slides with Mowiol^®^ 4–88. Imaging was performed on a Zeiss LSM 710 system with 40× oil immersion objective. Images were processed with ZEN lite software (Zeiss, Oberkochen, Germany).

#### 3.5.2. Determination of Antibacterial Efficacy against Intracellular *Mycobacterium tuberculosis*

MonoMac-6 human monocytes (2 × 10^5^ cells/1 mL medium/well) were cultured in RPMI-1640 medium containing 10 % FCS, in a 24-well plate, 24 h prior to the experiment. Adherent cells were infected with *Mycobacterium tuberculosis* H_37_Rv, at a multiplicity of infection (MOI) of 10. Nonphagocytosed extracellular bacteria were removed by washing the culture three times with serum-free RPMI. After 1 day of incubation, infected cells were treated with INH-conjugated Penetratin and Transportan 5, 10 and 50 μM final concentrations. For comparison, INH was also assayed at 100 μM concentration. As control, infected cells were treated with culture media. After 3 days, the treatment was repeated with fresh solutions of the compounds, and cells were incubated for an additional 3 days. After 3 washing steps, infected cells were lysed with 2.5 % sodium dodecyl sulphate (SDS) solution, and 100 μL lysate was transferred to L-J medium (BBL Löwenstein–Jensen medium, Beckton Dickinson). Colony-forming units (CFU) were enumerated after 4 weeks of incubation, and the number of bacteria was calculated by using a standard dilution series of *M. tuberculosis* [60].

## 4. Conclusions

INH-conjugated peptides with various composition, hydrophobicity and charge character were compared in an experimental membrane affinity study, using lipid monolayer models. In most cases, increased membrane affinity was observed, especially with mycolic-acid-containing lipid layer induced by the conjugation with INH. Hydrophobic character of the peptides seems to favor the interaction with lipid layers within the set of cationic peptides studied here. CD spectroscopic measurement allowed for the analysis of the correlation between the secondary structural change of the peptides and their INH-conjugates and the membrane affinity. The penetration into the lipid layer was in line with the destruction of ordered structure of lipid layer observed visually by AFM in nanometric range.

In vitro assays supported the cellular uptake and antibacterial effect of the INH–peptide conjugates; furthermore, an important difference in the intracellular localization was also revealed, depending on the carrier peptide. Nuclear translocation of Penetratin and its ability to carry various chemically conjugated cargos within the nucleus has been reported so far [56], and this feature made Penetratin a good candidate to utilize in gene therapy [61,62]. Our recent study added a supplement feature to Penetratin as a carrier molecule, namely the use as vector for peptide delivery of antitubercular agents and utilization of it against intracellular bacteria.

## Figures and Tables

**Table 1:** Analytical characterization of the peptides and INH–peptide conjugates studied in this work.

Compound ^a^	Sequence	*M* ^b^	*R*_t_^c^	*H* ^d^	*Z* ^e^
OT20	TKPKGTKPKGTKPKGTKPKG	2062.3	6.7	1.1	9+
Crot(1–9,38–42)	YKQCHKKGGKKGSG	1503.8	6.8	0.8	6+
Buforin II (5–21)	RAGLQFPVGRVHRLLRK	2001.2	10.9	0.2	6+
Penetratin	RQIKIWFQNRRMKWKK	2244.3	10.9	0.5	8+
Dhvar4	KRLFKKLLFSLRKY	1838.2	11.8	0.3	7+
Magainin II	GIGKFLHSAKKFGKAFVGEIMNS	2464.3	12.8	−0.1	4+
CM15	KWKLFKKIGAVLKVL	1769.2	13.7	−0.1	6+
Transportan	AGYLLGKINLKALAALAKKIL	2180.4	16.8	−0.3	5+
INH–OT20	INH-TKPKGTKPKGTKPKGTKPKG	2239.3	7.0	-	8+
INH–Crot(1–9,38–42)	INH-YKQCHKKGGKKGSG	1680.9	7.3	-	5+
INH–Buforin II (5–21)	INH-RAGLQFPVGRVHRLLRK	2178.3	11.0	-	5+
INH–Penetratin	INH-RQIKIWFQNRRMKWKK	2421.4	11.5	-	7+
INH–Dhvar4	INH-KRLFKKLLFSLRKY	2015.2	12.8	-	6+
INH–Magainin II	INH-GIGKFLHSAKKFGKAFVGEIMNS	2641.4	13.3	-	3+
INH–CM15	INH-KWKLFKKIGAVLKVL	1946.2	14.3	-	5+
INH–Transportan	INH-AGYLLGKINLKALAALAKKIL	2357.5	16.9	-	4+

^a^ All peptides and conjugates were amidated on the *C*-terminus and isolated as TFA salts. ^b^ Exact mass measured on a Thermo Scientific Q Exactive™ Focus Hybrid Quadrupole-Orbitrap™ Mass Spectrometer. ^c^ Retention time on a YMC-Pack ODS-A C18 (5 µm, 120 Å) 150 × 4.6 mm column, gradient: 5% B, 2 min; 5%–100% B, 20 min. ^d^ Hydrophilicity was calculated by using values of amino acids expressing hydrophilicity of each amino acid [36]. ^e^ Net charge at neutral pH.

**Table 2:** Roughness values, *R*_a_, *R*_q_ and *R*_z_ characterizing the morphology of lipid layers and penetrated lipid layers determined by AFM imaging.

Peptide	Lipid System	*R*_a_/nm	*R*_q_/nm	*R*_z_/nm
	DPPC	0.10	0.13	0.36
	DPPC+mycolic acid	0.25	0.33	1.08
INH–Penetratin	DPPC	0.21	0.27	0.92
	DPPC+mycolic acid	0.35	0.45	1.33
INH–Transportan	DPPC	0.23	0.37	0.90
	DPPC+mycolic acid	0.59	0.94	2.40
